# *APOE* and *MAPT* Are Associated With Dementia in Neuropathologically Confirmed Parkinson's Disease

**DOI:** 10.3389/fneur.2021.631145

**Published:** 2021-02-05

**Authors:** Jon-Anders Tunold, Hanneke Geut, J. M. Annemieke Rozemuller, Sandra Pilar Henriksen, Mathias Toft, Wilma D. J. van de Berg, Lasse Pihlstrøm

**Affiliations:** ^1^Department of Neurology, Oslo University Hospital, Oslo, Norway; ^2^Institute of Clinical Medicine, University of Oslo, Oslo, Norway; ^3^Section Clinical Neuroanatomy and Biobanking, Department of Anatomy and Neurosciences, Amsterdam UMC, Location Vrije Universiteit Amsterdam, Amsterdam Neuroscience, Amsterdam, Netherlands; ^4^Department of Pathology, Amsterdam UMC, Location Vrije Universiteit Amsterdam, Amsterdam Neuroscience, Amsterdam, Netherlands

**Keywords:** parkinson's disease, dementia, neuropathology, genetics, association study, APOE, MAPT

## Abstract

**Introduction:** Cognitive decline and dementia are common and debilitating non-motor phenotypic features of Parkinson's disease with a variable severity and time of onset. Common genetic variation of the Apolipoprotein E (*APOE*) and micro-tubule associated protein tau (*MAPT*) loci have been linked to cognitive decline and dementia in Parkinson's disease, although studies have yielded mixed results. To further elucidate the influence of *APOE* and *MAPT* variability on dementia in Parkinson's disease, we genotyped postmortem brain tissue samples of clinically and pathologically well-characterized Parkinson's donors and performed a survival analysis of time to dementia.

**Methods:** We included a total of 152 neuropathologically confirmed Parkinson's disease donors with or without clinical dementia during life. We genotyped known risk variants tagging the *APOE* ε4 allele and *MAPT* H1/H2 inversion haplotype. Cox proportional hazards regression analyses adjusted for age at onset, sex and genetic principal components were performed to assess the association between the genetic variants and time from motor onset to onset of dementia.

**Results:** We found that both the *APOE* ε4 allele (HR 1.82, 95 % CI 1.16–2.83, *p* = 0.009) and *MAPT* H1-haplotype (HR 1.71, 95 % CI 1.06–2.78, *p* = 0.03) were associated with earlier development of dementia in patients with Parkinson's disease.

**Conclusion:** Our results provide further support for the importance of *APOE* ε4 and *MAPT* H1-haplotype in the etiology of Parkinson's disease dementia, with potential future relevance for risk stratification and patient selection for clinical trials of therapies targeting cognitive decline in Parkinson's disease.

## Introduction

Parkinson's disease (PD) is a heterogenous disorder in terms of clinical presentation and rate of progression. Dementia is one of the most debilitating non-motor manifestations of the disease, with broad implications for both patients and caregivers (1–3). Longitudinal studies have shown that most patients ultimately develop Parkinson's disease dementia (PDD) if they survive long enough, although the time of onset is highly variable (4, 5). Cognitive disability is not only a feature of advanced disease, as 36% of patients meet criteria for mild cognitive impairment already at clinical diagnosis (6) and 17% of patients develop dementia within five years from disease onset (7). Identification of biomarkers, including common genetic variants predicting early cognitive decline and dementia, could provide important insights into the biological and molecular underpinnings of PDD, benefit recruitment to clinical trials and identify potential targets for novel therapeutics.

Genome-wide association studies (GWAS) have identified genetic susceptibility loci for sporadic PD, with the latest meta-analysis bringing the number up to 90 risk signals across 78 loci (8). Genetic variability may not only affect the risk of developing PD, but also influence the clinical course of the disease. Several genetic loci have been hypothesized as risk factors for dementia in sporadic PD, among them *APOE* and *MAPT*, showing partly conflicting results in previously published reports (9).

Coding variation in *APOE* on chromosome 19 gives rise to three common alleles: ε2, ε3, and ε4. The *APOE* ε4 allele is a strong and well-established genetic risk factor for Alzheimer's disease (AD) (10), and the top GWAS signal in dementia with Lewy bodies (DLB) (11). While *APOE* does not seem to alter the risk for PD in itself according to GWAS results, the ε4 allele has been studied as a potential risk factor for cognitive decline and development of dementia in PD patients, with several larger studies reporting a significant association (12, 13).

An inversion polymorphism on chromosome 17q21, containing *MAPT* and several other genes, gives rise to the H1 and H2 haplotypes in European populations (14). Single-nucleotide polymorphisms (SNPs) tagging the H1-haplotype have consistently been among the most significant association signals in GWAS of PD-risk (8, 15, 16). The *MAPT* gene encodes the tau protein that is found to aggregate in neurofibrillary tangles (NFT), a core neuropathological feature of AD, but also found in varying degrees in PD and PDD patients upon autopsy (17, 18). Interestingly, the *MAPT* H1-haplotype has also been reported to be associated with an accelerated rate of cognitive decline and earlier development of dementia in PD patients (7, 19, 20), yet larger studies have not been able to replicate this finding (12, 21).

Discrepant results across previous genetic association studies of cognitive outcomes in PD could potentially arise from differences in methodology, in particular with respect to inclusion criteria, duration of follow-up and outcome measures used to assess cognitive decline. A study based on brain bank samples can take advantage of gold standard diagnostics and clinical data that cover the patients' entire lifespan. In this study, we investigated the association of SNPs in the *APOE* and *MAPT* loci with time to dementia by retrospective survival analysis in neuropathologically defined PD brain donors.

## Methods

### Subjects

All subjects were neuropathologically confirmed patients with PD or PDD from the Netherlands Brain Bank (NBB, www.brainbank.nl). All brains available from the NBB from 1989 to 2017 (*n* = 3,853) were considered for study inclusion according to the selection criteria. Written, informed consent for the use of clinical information and tissue samples for research purpose, was collected from the donors or their next of kin.

Standardized brain autopsies and neuropathological examinations were performed by experienced neuropathologists (AR and WB). Neuropathological assessment of Lewy Body (LB)-related α-synuclein pathology was done according to BrainNet Europe guidelines (22) and assessment of AD neuropathologic change was done according to National Institute on Aging-Alzheimer's Association (NIA-AA) guidelines (23).

Clinical information was extracted from the medical records provided by the NBB. The diagnosis of PD was based on the combination of the clinical syndrome of PD [UK Parkinson's Disease Society Brain Bank criteria (24)], and moderate to severe loss of neurons in the substantia nigra in association with Lewy pathology in at least the brainstem with or without limbic and cortical brain regions (25). When dementia had been diagnosed during life, donors fulfilling these criteria were classified as PDD. A diagnosis of dementia was made during life by a neurologist or geriatrician, or retrospectively based on neuropsychological test results showing disturbances in at least two core cognitive domains (26) or Mini-Mental State Examination (MMSE) score <20. Distinction between DLB and PDD was made based on the 1-year rule, where dementia presenting before or within 1 year of parkinsonism onset was diagnosed as DLB, and not included in this study (27). Cases diagnosed as having both PD and AD were also excluded from the study.

### Genotyping

DNA was extracted from brain tissue. Genotyping was carried out on the Infinium® NeuroChip Consortium Array (Illumina, San Diego, CA USA) (28). Quality control was carried out in PLINK version 1.9 (29). Samples passing standard quality control, including filtering of variants and individuals based on call rate (< 0.95), Hardy-Weinberg equillibrium (*p* < 0.000001), relatedness (pi-hat > 0.125), excess heterozygosity (> 4SD from mean), sex-check and ancestry assessed by principal component plots, were imputed using the Michigan Imputation Server (30). We selected rs1800547 to discriminate between the *MAPT* H1 and H2 haplotypes, and used rs429358 and rs7412 to define the *APOE* ε2, ε3, and ε4 alleles as previously described (31, 32).

The NeuroChip array was also used to screen for known pathogenic mutations in relevant Mendelian PD genes. Covering the majority of definitely and probably pathogenic variants in the autosomal dominant genes *SNCA, LRRK2*, and *VPS35*, we identified no mutation carriers (Supplementary Table 1).

### Statistical Analysis

All statistical analyses were carried out in R (version 4.0.2; http://www.r-project.org). Differences in baseline demographics and clinical variables between patients with PD and PDD were assessed using *t*-tests for continuous variables and chi-square tests for categorical variables. Ordinal variables (neuropathological scores) were compared using the Wilcoxon Rank Sum Test, while associations between neuropathology and genotypes were measured by odds ratios using ordinal logistic regression adjusting for age at death and sex. For the survival analysis we used the R package “survival.” Cox proportional hazards regression models were employed to assess the relationship between genotype and dementia onset. The event variable was presence of dementia. As time variable we used disease duration at dementia onset for PDD and disease duration at death for PD. Separate analyses were carried out for each risk locus, with sex, age at motor symptom onset and the first five genetic principal components as covariates. We estimated hazard ratio (HR) and the 95% confidence interval (CI). P values for each covariate were obtained from the Wald test. The results were visualized as Cox regression-adjusted curves using the R package “survminer.” A combined plotting and testing approach was employed to check the proportional hazards assumptions. A *p* < 0.05 was used as significance threshold in this study.

## Results

One hundred sixty five donors (PD *n* = 79 and PDD *n* = 86) were identified. A total of 13 cases were excluded for missing clinical, neuropathological or genotype data, or failing quality control. A total of 152 cases (PD *n* = 71 and PDD *n* = 81) meeting clinical and neuropathological criteria were included in the final analysis. The demographic and clinical characteristics are displayed in Table 1. There were no significant differences in sex distribution, age at disease onset, disease duration or age at death between PD and PDD patients.

**Table 1:** Clinical characteristics of cases with Parkinson's disease non-demented (PDnD) and Parkinson's disease dementia (PDD).

	**PDnD**	**PDD**	***p***
	***N* = 71**	***N* = 81**	
Sex, male (%)	43 (60.6)	57 (70.4)	0.271
Age at disease onset, mean (SD)	61.3 (13.0)	64.2 (9.5)	0.117
Age at dementia onset, mean (SD)	-	73.7 (7.0)	-
Disease duration, mean (SD)	15.5 (7.7)	13.6 (6.7)	0.102
Motor dementia interval, mean (SD)	-	9.4 (5.8)	-
Dementia duration, mean (SD)	-	4.1 (2.8)	-
Age at death, mean (SD)	77.0 (9.3)	77.8 (6.5)	0.515

*SD: standard deviation. P value from t-tests for continuous variables and chi-square tests for categorical variables (sex)*.

**Figure 1:**
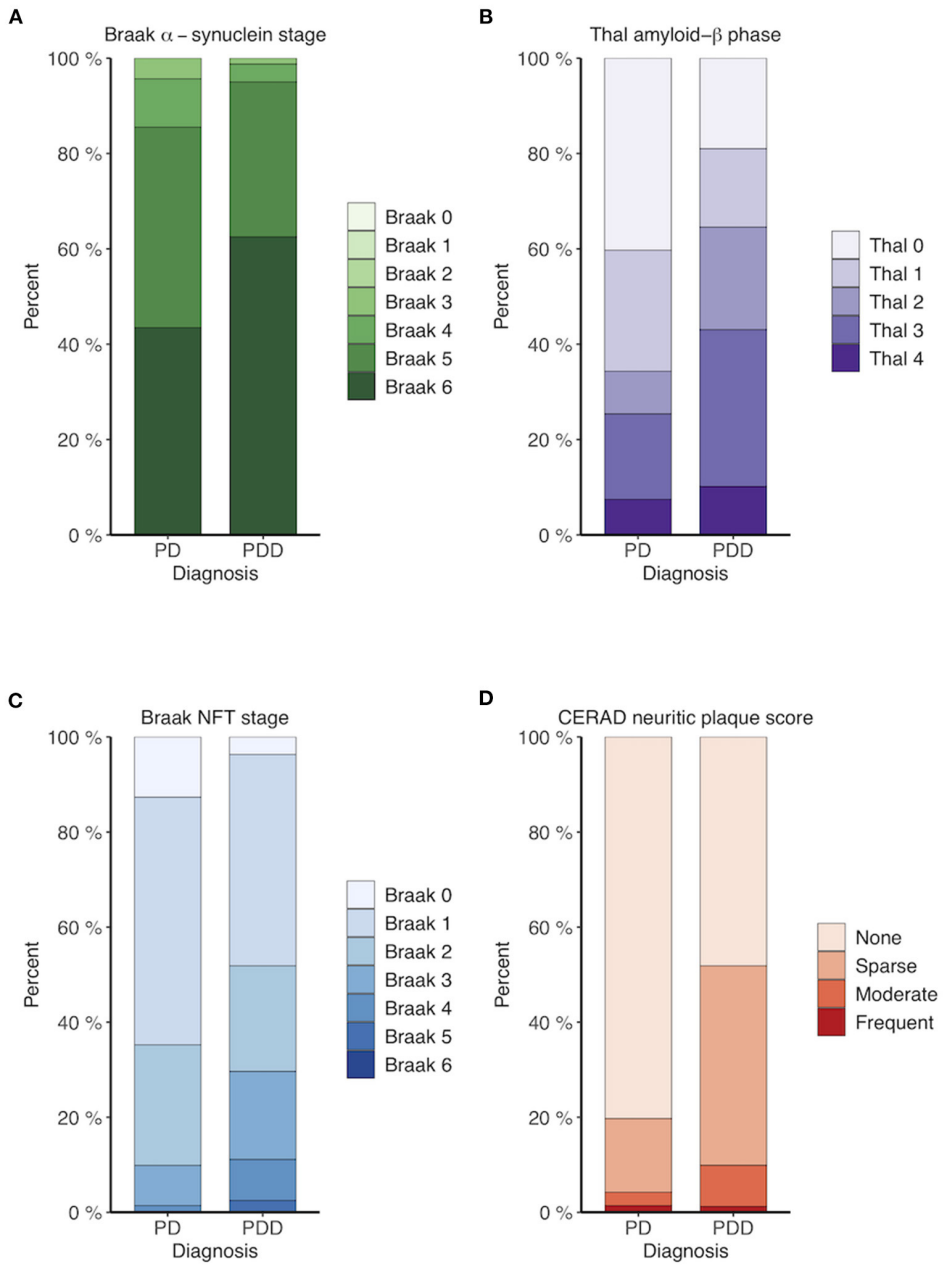
Neuropathological scores for PD and PDD patients. **(A)** Braak α-synuclein stage. **(B)** Thal amyloid-β phase. **(C)** Braak neurofibrillary tangle (NFT) stage. **(D)** CERAD neuritic plaque score. PDD patients display more advanced LB, Aβ, and tau pathology compared to PD patients.

Braak α-synuclein stage (*p* = 0.01), Thal amyloid-β (Aβ) phase (*p* = 0.001), Braak NFT stage (*p* = 0.003) and CERAD neuritic plaque score (*p* < 0.001) were all higher in PDD compared to PD patients (Figure 1 and Supplementary Table 2). Applying the NIA-AA criteria, intermediate or high AD co-pathology was present in 7% (5 of 67) of PD patients and 14% (11 of 80) of PDD patients. *APOE* ε4 was significantly associated with Thal Aβ phase (OR 4.85, *p* < 0.001) and CERAD neuritic plaque score (OR 4.97, *p* < 0.001), but not Braak NFT or Braak α-synuclein stage (Supplementary Table 3). The *MAPT* H1-haplotype was not significantly associated with any of the neuropathological scores.

**Figure 2:**
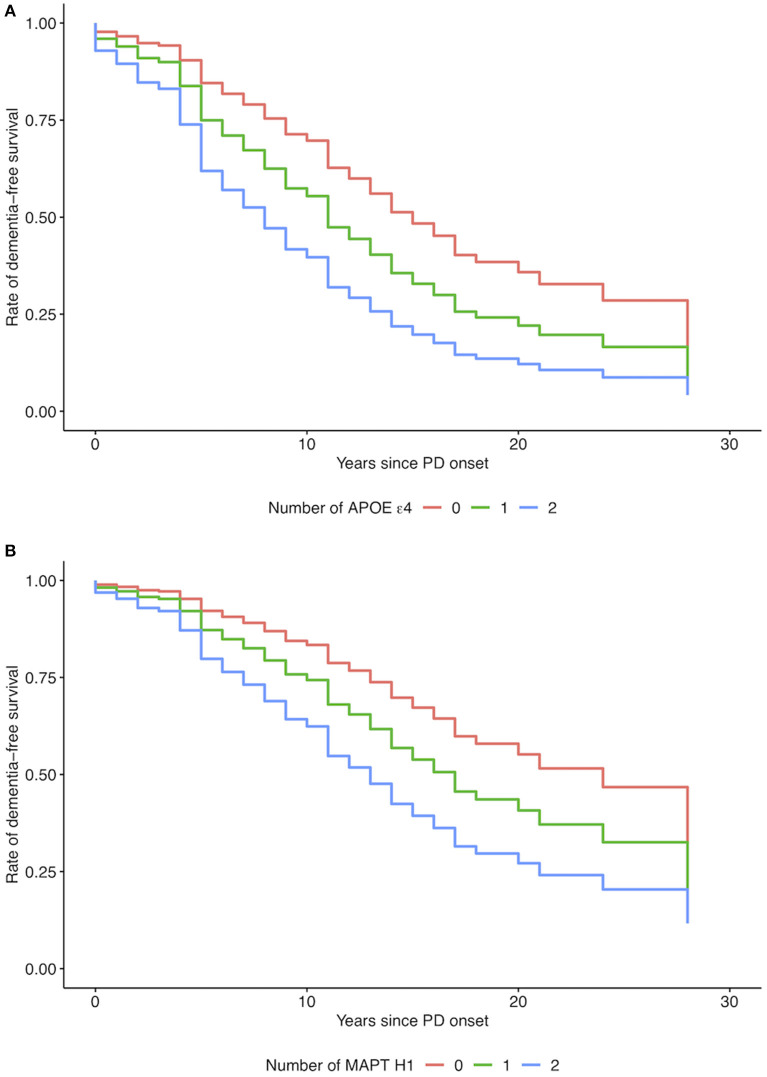
Adjusted survival curves for Cox proportional hazards model. **(A)** Apolipoprotein E (*APOE*) ε4 (0 = negative, 1 = ε4 heterozygous, 2 = ε4 homozygous), and **(B)** Microtubule-associated protein tau (*MAPT*) H1-haplotype (0 = negative, 1 = heterozygous, 2 = homozygous).

In the Cox proportional hazards model the *APOE* ε4 allele was significantly associated with a shorter time between PD onset and diagnosis of PDD (HR per ε4 allele 1.82, 95 % CI 1.16–2.83, *p* = 0.009, Table 2 and Figure 2A). When Thal Aβ phase or CERAD neuritic plaque score were added as covariates, the association with time to dementia was no longer significant (*p* = 0.23 and *p* = 0.11, respectively). The *MAPT* H1-haplotype was also significantly associated with a shorter time to dementia (HR per H1 haplotype 1.71, 95% CI 1.06–2.78, *p* = 0.03, Table 2 and Figure 2B). Later age at onset was significantly associated with shorter time to dementia in both models (HR 1.09, 95% CI 1.06–1.12, *p* < 0.001).

**Table 2:** Risk variant frequencies and results from Cox proportional hazards regression models with age at onset, sex, and genetic principal components as covariates.

**Variant**	**Frequency**	**HR**	**95% CI for HR**	***p***
APOE ε4	PDnD: 0.11	1.82	1.16–2.83	0.009^*^
	PDD: 0.14			
MAPT H1/H1	PDnD: 0.68	1.71	1.06–2.78	0.03^*^
	PDD: 0.77			

*APOE, Apoliporotein E; HR, hazard ratio; CI, confidence interval; MAPT, microtubule-associated protein tau*.

* *P value from the Wald test*.

## Discussion

In this study we explored the genetic effects of *MAPT* and *APOE* on onset of dementia in PD in a neuropathologically characterized cohort. With the advantages of definite diagnosis and clinical data from the patients' entire lifespan, we found that even in a small sample, both the *APOE* ε4 allele and the *MAPT* H1-haplotype were significantly associated with an accelerated onset of dementia in PD patients.

Several studies have examined the effects of *APOE* ε4 on cognitive decline and dementia in PD. Many of these have had cross-sectional design, and while some have demonstrated an association with *APOE* ε4 and lower cognitive performance (21), others have failed to do so (33). Consistent with our results, a previous study of PD patients demonstrated earlier development of dementia among *APOE* ε4-carriers (HR 1.90, 95% CI 1.05–3.44) (34). In line with our data, two recent meta-analyses reported an increased risk of dementia in PD patients who carried the *APOE* ε4 allele, although regional differences in effect size were noted (35, 36). Longitudinal studies have found associations with *APOE* ε4 and a more rapid cognitive decline measured on both screening instruments for global cognition (37, 38) and battery-style assessment of mental status (12, 39). In a recent GWAS on PD progression using longitudinal data from three large cohorts, the top hit for cognitive progression was rs429358 tagging *APOE* ε4 (40). In contrast, variants in the *APOE*-gene were not associated with cognitive decline or dementia at 3.5, 5, or 10 year follow-up in the CamPaIGN study, a UK incident cohort of PD patients (7, 20), or with shorter time to dementia in another longitudinal study (41). While longitudinal designs represent a gold standard for tracking disease progression, they may be hampered by small sample size, short follow-up time and loss to follow-up. Taken together, the weight of evidence favors an effect of *APOE* on cognitive decline and dementia in PD, further supported by our results.

We also found a significant association between *MAPT* H1 and time to dementia in PD. This locus is less established than *APOE* in the previous literature on genetic risk factors of cognitive progression. The CamPaIGN study was the first to report an association between the *MAPT* H1/H1 genotype and cognitive decline in PD (19). The results were confirmed in the subsequent 5- and 10-year follow-up studies, supporting the *MAPT* H1/H1 genotype as predictive of dementia (7, 20). The association between *MAPT* genotype and PDD has later been replicated (42), while other studies have failed to do so (12, 21, 38). Contrary to our results, no association between *MAPT* H1/H1 genotype and dementia onset was found in a previous survival analysis of 298 PD patients where 59 progressed to dementia (34). A prospective investigation of 212 patients noted associations between *MAPT* H1 and specific cognitive outcome measures, but not with the overall rate of cognitive decline (12). The authors of this study hypothesized that the significant signal reported in the CamPaIGN study could represent an effect specific to early dementia development, as the CamPaIGN patients were included at diagnosis and assessed for progression to PDD at 3 years. Our data do not support this explanation of previously discrepant results, as the mean disease duration at dementia onset in the PDD group was 9–10 years in our study.

The underlying mechanisms linking *APOE* and *MAPT* variants to dementia are unclear, however neuropathological studies suggests that protein aggregation is pivotal in this association. In our study *APOE* ε4 was significantly associated with both Thal Aβ phases and CERAD neuritic plaque scores, supporting that *APOE* ε4 exerts its genetic risk on dementia primarily through Aβ neuropathology. The *MAPT* H1 haplotype was not associated with any neuropathological scores in our study. Concomitant AD pathology (Aβ plaques and NFT) is found in variable amounts upon autopsy in PD and PDD brains, and is more prevalent in PDD compared to PD (17, 43, 44). This is indeed true for our cases, as neuropathological examination revealed significantly more advanced Thal Aβ phases, Braak NFT stages and CERAD neuritic plaque scores in PDD compared to PD samples.

Several lines of evidence support the role of cortical LB pathology as the major pathological driver of dementia in PD (17, 45), and in our study PDD donors had significantly more advanced Braak α-synuclein stages than PD donors. While it seems likely that *APOE* ε4 mediates dementia through an Aβ-dependent pathway, previous studies have also reported an effect of *APOE* ε4 on cognitive outcome and severity of cortical LB pathology in patients with low concomitant AD-pathology (46, 47). Corroborating these findings, two recent experimental studies have shown evidence that *APOE* ε4 may promote LB pathology independent of Aβ pathology (48, 49). In our results, however, the association with dementia was dependent on Aβ, as the signal was no longer significant when adjusting for Thal Aβ phase or CERAD neuritic plaque score.

While the presence of tau pathology has been correlated with reduced time to dementia (50), some evidence also supports that the *MAPT* H1-haplotype may influence the cortical LB burden (51), suggesting *MAPT* also may promote dementia in more than one way. This idea was not supported by our data, but we note that the size of our study provided limited statistical power to disentangle potentially complex correlations between genotype and various neuropathologies. We also acknowledge that although the H1 inversion haplotype on chromosome 17 is commonly named after *MAPT*, it contains a number of other genes, and the mechanism driving the association signal for PD risk has yet to be unequivocally established. Recent evidence suggest that rather than *MAPT*, the disease-relevant gene could be the neighboring *KANSL1*, which is involved in autophagy regulation (52).

The clinical diagnosis of PD can be challenging, with a diagnostic accuracy of 80.6% when pathological examination is used as the gold standard (53). The strength of this study lies in the neuropathological confirmation of diagnosis and the retrospective overview of the clinical disease course from the patients' entire lifespan. Some limitations of our study should be noted. First, clinical information was obtained by retrospective review of medical records posing a risk for information bias, in particular regarding approximation of timing of events. However, the timing of motor symptom onset and dementia onset observed in this study harmonize well with previous reports (17, 54). Second, we acknowledge that lack of extensive neuropsychological evaluation is a limitation. In theory, death and dementia may be competing events and potentially bias the estimated effect of genotypes on dementia development. *APOE* ε4 has been associated with decreased longevity, but we observed similar age at death in PD and PDD, and any theoretical bias from this effect would skew results in the opposite direction of our findings (55). Further corroboration of the genetic associations reported here is warranted, preferably in longitudinal cohorts. Third, given the limited sample size and statistical power of our study, we narrowly selected only two candidate loci among several previously reported as associated with cognition in PD. A broader perspective on the genetic architecture of PDD would have to consider the contribution from loci such as *SNCA, GBA, COMT* and potentially others (9), and ideally also the possibility of synergistic interactions between these.

In conclusion, our study adds to the growing evidence supporting the role for not only *APOE* ε4 but also the *MAPT* H1 haplotype in development of dementia in PD. Detecting significant associations in a small, but well-characterized neuropathological sample, we anticipate that larger genetic association studies of neuropathological phenotypes will be a fruitful strategy to further disentangle molecular mechanisms in neurodegenerative disorders. Ultimately, a better understanding of genotype-phenotype correlations may facilitate precision medicine in PD, improving risk prediction and patient stratification for novel targeted therapies.

## Data Availability Statement

The raw data supporting the conclusions of this article will be made available by the authors, without undue reservation.

## Ethics Statement

The studies involving human participants were reviewed and approved by Regional Committees for Medical and Health Research Ethics, Norway. The patients/participants provided their written informed consent to participate in this study.

## Author Contributions

J-AT performed statistical analyses and drafted the manuscript. HG and JR contributed clinical and neuropathological data. SH contributed to genotyping. MT contributed to study design and organized the study. WB contributed clinical and neuropathological data, contributed to study design and organized the study. LP designed and organized the study and contributed to genotyping, data analyses and drafting of the manuscript. All authors took part in critical revision of the manuscript and approved the submitted version.

## Conflict of Interest

The authors declare that the research was conducted in the absence of any commercial or financial relationships that could be construed as a potential conflict of interest.
