# Supplementary material for: APOE and MAPT Are Associated With Dementia in Neuropathologically Confirmed Parkinson's Disease
Source: Front Neurol. 2021 Feb 5;12:631145. doi: 10.3389/fneur.2021.631145 (PMC7892776; doi:10.3389/fneur.2021.631145)
Supplement: Supplementary file 1 [file Table_1.DOCX]

**Supplementary tables to: *APOE* and *MAPT* are associated with dementia in neuropathologically confirmed Parkinson’s disease**

**Supplementary table 1**

| **b37 position** | **Nucleotide change** | **Gene** | **dbSNP ID** | **Pathogenicity MDSGene** | **Successfully screened by NeuroChip** |
| --- | --- | --- | --- | --- | --- |
| 4:90749299 | c.158C>A | SNCA | rs542171324 | Definitely |  |
| 4:90749300 | c.157G>A | SNCA | rs104893877 | Definitely | Yes |
| 4:90749305 | c.152G>A | SNCA | rs431905511 | Definitely | Yes |
| 4:90749321 | c.136G>A | SNCA | rs104893875 | Probably | Yes |
| 4:90756731 | c.88G>C | SNCA | rs104893878 | Definitely | Yes |
| 12:40657677 | c.1630A>G | LRRK2 | rs79996249 | Probably | Yes |
| 12:40677699 | c.2264C>T | LRRK2 | rs34410987 | Probably | Yes |
| 12:40689268 | c.2918G>A | LRRK2 | rs75148313 | Probably | Yes |
| 12:40702420 | c.4111A>G | LRRK2 | rs17466213 | Probably | Yes |
| 12:40703027 | c.4309C>A | LRRK2 | rs74163686 | Definitely |  |
| 12:40704236 | c.4321C>G | LRRK2 | rs33939927 | Definitely |  |
| 12:40704236 | c.4321C>T | LRRK2 | rs33939927 | Definitely | Yes |
| 12:40704237 | c.4322G>A | LRRK2 | rs34995376 | Definitely |  |
| 12:40704306 | c.4522A>G | LRRK2 | rs1040765034 | Probably |  |
| 12:40704439 | c.4449C>G | LRRK2 | rs756714261 | Probably | Yes |
| 12:40714916 | c.5096A>G | LRRK2 | rs35801418 | Definitely | Yes |
| 12:40715840 | c.5174G>A | LRRK2 | rs72547979 | Probably | Yes |
| 12:40716270 | c.5467C>A | LRRK2 | rs72547980 | Probably | Yes |
| 12:40717058 | c.5606T>C | LRRK2 | rs35602796 | Probably | Yes |
| 12:40734122 | c.5971A>G | LRRK2 |  | Probably |  |
| 12:40734182 | c.6035T>C | LRRK2 | rs34015634 | Probably | Yes |
| 12:40734202 | c.6055G>A | LRRK2 | rs34637584 | Definitely | Yes |
| 12:40734206 | c.6059T>C | LRRK2 | rs35870237 | Definitely | Yes |
| 12:40745484 | c.6523G>C | LRRK2 | rs776801536 | Probably |  |
| 12:40757242 | c.7067C>T | LRRK2 | rs113511708 | Probably | Yes |
| 12:40758750 | c.7315C>A | LRRK2 | rs201913693 | Probably |  |
| 16:46696364 | c.1858G>A | VPS35 | rs188286943 | Definitely | Yes |
| 16:46702919 | c.1570C>T | VPS35 | rs184277092 | Probably | Yes |
| 16:46708541 | c.946C>T | VPS35 | rs770029606 | Probably |  |

The table lists all definitely or probably pathogenic mutations in autosomal dominant PD listed in the MDSGene database (<https://www.mdsgene.org/>) and whether the variant was successfully genotyped on the NeuroChip. No mutations were detected.

**Supplementary table 2**

|  | **PD**  N =71 | **PDD**  N = 81 | **p value** |
| --- | --- | --- | --- |
| Braak α-synuclein stage, median (range) | 5 (3-6) | 6 (3-6) | 0.01 |
| Thal amyloid-β phase, median (range) | 1 (0-4) | 2 (0-4) | 0.001 |
| Braak NFT stage, median (range) | 1 (0-4) | 2 (0-5) | 0.003 |
| CERAD score, median (range) | 0 (0-3) | 1 (1-3) | < 0.001 |

Neuropathological stages for PD and PDD donors. P value from Wilcoxon Rank Sum Test. For convenience CERAD score was converted to numeric variables (none = 0, sparse = 1, moderate = 2, frequent = 3).

**Supplementary table 3**

| **Variant** | **Neuropathology score** | **OR** | **95 % CI for OR** | **p value** |
| --- | --- | --- | --- | --- |
| ***APOE* ε4** | Braak α-synuclein stage | 1.79 | 0.91-3.67 | 0.10 |
|  | Thal amyloid-β phase | 4.85 | 2.48-9.70 | < 0.001 |
|  | Braak NFT stage | 1.37 | 0.71-2.68 | 0.35 |
|  | CERAD score | 4.97 | 2.48-10.29 | < 0.001 |
| ***MAPT* H1** | Braak α-synuclein stage | 0.93 | 0.48-1.76 | 0.82 |
|  | Thal amyloid-β phase | 1.70 | 0.94-3.16 | 0.09 |
|  | Braak NFT stage | 1.14 | 0.62-2.11 | 0.67 |
|  | CERAD score | 1.41 | 0.71-2.93 | 0.34 |

Association between APOE and MAPT genotypes, and neuropathological scores. OR, odds ratio; CI, confidence interval.
